# Role of Intraoperative Biopsy and Postoperative Upper Gastrointestinal Endoscopy in Patients Undergoing Surgery for Perforated Gastric Ulcer: A Retrospective Study

**DOI:** 10.7759/cureus.77092

**Published:** 2025-01-07

**Authors:** Madalena Trindade, Daniel Gomes Pinto, Nuno Carvalho, Carlos Luz

**Affiliations:** 1 Department of Surgery, Unidade Local de Saúde de Almada-Seixal, Almada, PRT; 2 Department of Pathology, Unidade Local de Saúde de Almada-Seixal, Almada, PRT

**Keywords:** gastric cancer (gc), gastric ulcer perforation, intra-operative biopsy, ulcer biopsy, upper gastrointestinal endoscopy (ogd)

## Abstract

Introduction

The intraoperative biopsy of a perforated gastric ulcer is a common practice; however, it may prove to be therapeutically futile, as an upper gastrointestinal endoscopy (UGE) is indicated regardless of the result.

Objective

To evaluate the histology of the perforated gastric ulcer biopsy (PGUB) and its effect on the subsequent clinical course.

Methods

A retrospective study (May 2017 to June 2024) of patients diagnosed with perforated gastric ulcer (K251 and K255 - ICD-10), with a review of medical records in the Medical Support System, was conducted. The histology of the PGUB, the performance of UGE, the degree of concordance between the histology of both, and the potential waiver of UGE were evaluated. Informed consent was waived, as this is an observational study without any interference with clinical conduct.

Results

During the evaluation period, 21 patients underwent PGUB, and 19 patients had a benign histological result; one patient was diagnosed with high-grade non-Hodgkin lymphoma, and one patient had gastric adenocarcinoma. In the following period, 10 patients underwent UGE, and in none of them did the histological diagnosis change. Of the remaining 11 patients who did not undergo UGE, five died, one was lost to follow-up, three are awaiting the procedure, and only two did not undergo the endoscopic exam.

Conclusion

The intraoperative biopsy of a perforated gastric ulcer is possibly futile, as it does not alter the clinical course. Regardless of the histological result obtained, a UGE is requested.

## Introduction

Peptic ulcer disease (PUD) is a common condition, with an estimated prevalence of 5% to 10% during a lifetime. Perforation of peptic ulcer (PPU) is often the primary cause of emergency surgery in patients with PUD, occurring with a mortality rate of 23% [1]. The clinical picture and treatment of PPU were first described by Mikulicz-Radecki in the late 19th century [2].

In recent decades, the number of patients with PPU has decreased, thanks to the use of proton pump inhibitors and improved endoscopic techniques [3]. Furthermore, the understanding of the pathogenesis of PUD, related to *Helicobacter pylori* infection and the overuse of non-steroidal anti-inflammatory drugs, has led to new forms of treatment and prevention [4].

Although rare, gastric cancer perforation is a severe complication, present in less than 3% of patients with gastric neoplasia. However, it represents 10% to 16% of patients who have gastric perforation [5]. Spontaneous gastric perforation should raise suspicion of neoplasia, as it may be the presenting form of the disease [6,7].

Intraoperative biopsy of gastric ulcer (IGUB) is routinely performed in patients with PPU for the early diagnosis of gastric cancer [4,8-12]. In the case of PPU, the malignancy rate ranges from 4% to 14%, and it is common practice to biopsy the edges or elliptically excise the lesion [13].

However, recently, there have been some authors questioning the purpose of this biopsy [9]. The availability of endoscopic techniques gives us another option for obtaining a histological exam of the ulcer, without the need for edge excision. Additionally, elliptical excision is not a harmless procedure. A recent German retrospective study concluded that patients who underwent elliptical excision had more morbidity than those who didn’t. They described higher rates of complications (Clavien-Dindo III or higher), such as suture dehiscence, postoperative bleeding, and surgical site infections. Moreover, the exclusion of malignancy could be expendable in the emergency setting when performing damage control surgery [14].

After urgent treatment and control of the PPU site, upper gastrointestinal endoscopy (UGE) is advised to assess the possible presence of malignancy. This follow-up is crucial to prevent the progression of potential gastric neoplasia [3]. We ought to know if there is malignancy underlying the PPU, but is the IGUB the best way to do it? The aim of this study is to evaluate the impact of IGUB on the clinical management of patients with PUD.

## Materials and methods

A retrospective study of 51 consecutive patients diagnosed with perforated gastric ulcer (K251 and K255 - ICD-10) from May 2017 to June 2024, who were submitted to surgery. Of these 51 patients, 21 underwent ulcer biopsy or elliptical excision (42%, 21/51) because of the surgeon's decision. Biopsy specimens were placed in formalin and submitted for routine histological examination. The clinical parameters of the patients were collected retrospectively. Also, the histology of IGUB, microbiological isolation, the performance of UGE, the degree of concordance between the histology of both, and the potential waiver of UGE were evaluated. There were no protocols established at our institution; the follow-up was decided by each surgeon.

## Results

A total of 21 patients were included in the study. There were 71% (15/21) male patients, and the remainder were female (6/21). The average age was 66 years, ranging from 40 to 84 years old. Three of the 21 patients (14%) had known PUD previously and were undergoing treatment with proton pump inhibitors. The patients' characteristics are summarized in Table 1.

**Table 1:** Clinical parameters Clinical parameters of both groups: patients submitted to PPU surgery without IGUB and patients submitted to PPU surgery and IGUB. PPU, Perforated Peptic Ulcer; IGUB, Intraoperative Gastric Ulcer Biopsy; ASA, American Society of Anesthesiologists

Clinical parameters	Patients submitted to PPU surgery without IGUB (n = 30)		Patients submitted to PPU surgery and IGUB (n = 21)
Sex (male)	73% (22/30)		71% (6/21)
Age	55.4		65.9
ASA > II	30% (9/30)		52% (11/21)

The anatomical location of the gastric ulcer in the group of patients submitted to IGUB, according to the Johnson Modified Classification, was as follows: one patient with a fundus ulcer (type IV), two with body ulcers (type I), eight with antrum ulcers (type III), and 10 with pre-pyloric ulcers (type III). All patients underwent urgent open surgery and presented with peritonitis.

The surgical techniques used varied according to the surgeon's choice: suture, Cellan-Jones omentoplasty, pyloroplasty and omentoplasty, or Graham patch. In some patients, partial gastric resection techniques were necessary. In Table 2, we describe the surgical techniques performed.

**Table 2:** Surgical techniques Surgical techniques used in both groups: patients submitted to PPU surgery without IGUB and patients submitted to PPU surgery and IGUB. PPU, Perforated Peptic Ulcer; IGUB, Intraoperative Gastric Ulcer Biopsy

Surgery	Patients submitted to PPU surgery without IGUB (n = 30)	Patients submitted to PPU surgery and IGUB (n = 21)
Suture	9	2
Suture + omentoplasty	14	9
Cellan-Jones omentoplasty	3	6
Pyloroplasty + omentoplasty	1	1
Graham patch	-	2
Atypical gastrectomy + omentoplasty	-	1
Y de Roux subtotal gastrectomy	1	-
Resection of gastrojejunal anastomosis	1	-
Antrectomy + gastrojejunostomy	1	-

All patients presented with peritonitis, and some had microbiological isolation, as decided by each surgeon. Bacterial isolation was the most common and expected for this sample. However, there was a non-negligible percentage of patients with fungi isolated. Six fungi were isolated, and one patient had yeast shapes in the ascitic fluid. These patients presented to the Emergency Department without recurrent healthcare contact for emergent pathology, so this was not what would be expected. As shown in Table 3, there were 14 microbial isolations in eight patients: six patients had sterile microbiological isolation, eight had some isolated microorganisms, and in the remaining patients, no fluid was collected.

**Table 3:** Isolated microorganisms Microorganisms were isolated in the ascitic fluid of the eight patients with positive isolation.

Isolated microorganisms	n
Escherichia coli	2
Enterococcus faecalis	1
Streptococcus viridans	2
Streptococcus anginosus	1
Klebsiella pneumoniae	1
Candida albicans	3
Candida glabrata	2
Candida tropicalis	1
Yeast shape	1

It was described in the operative report that five patients had an ulcer with a malignant appearance, but none were confirmed. A biopsy of the ulcer edges was performed in 19 patients, and in the remaining two, an elliptical excision was performed.

**Figure 1:**
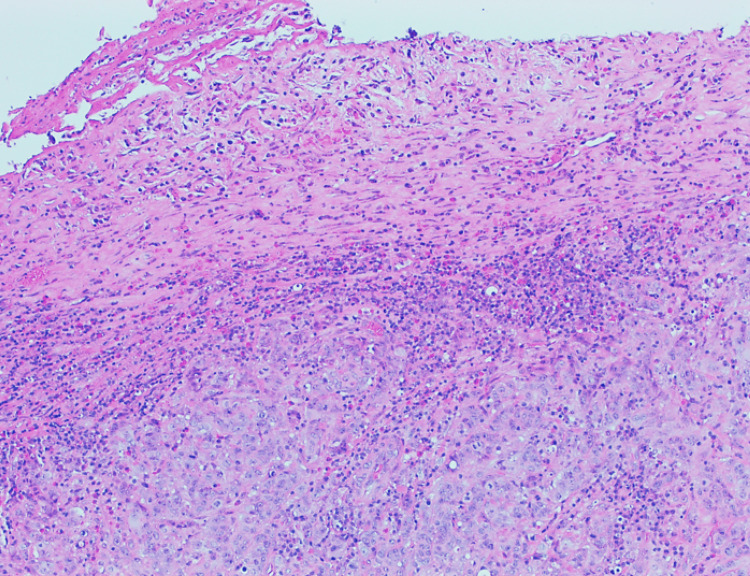
Gastric antrum: ulcer with underlying tubular adenocarcinoma (hematoxylin & eosin)

**Figure 2:**
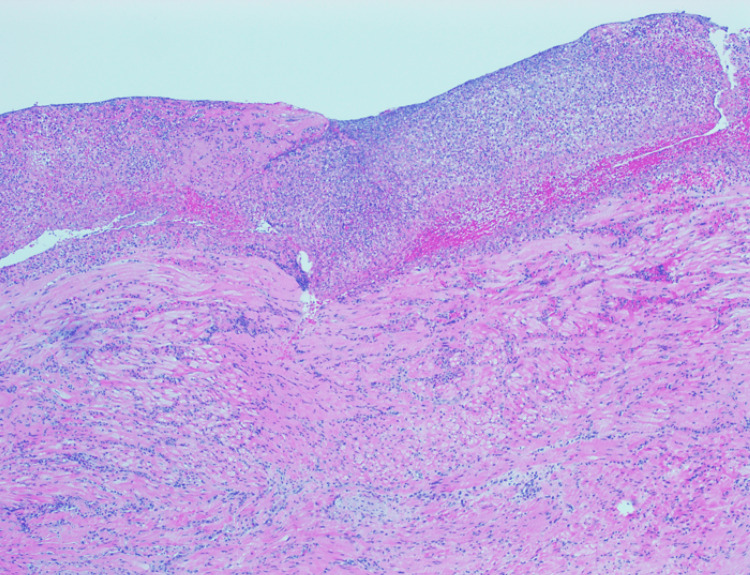
Gastric antrum: deep ulcer, touching the muscularis propria, with exhuberant fibrogranulocitary exsudate (hematoxylin & eosin)

**Figure 3:**
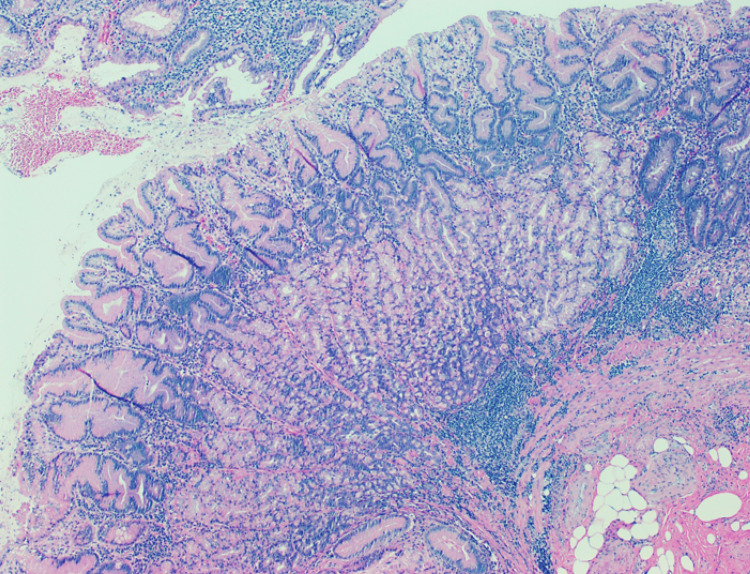
Gastric corpus: superficial chronic, band-like, inflammation, highly suggestive of Helicobacter pylori gastrites (hematoxylin & eosin)

**Figure 4:**
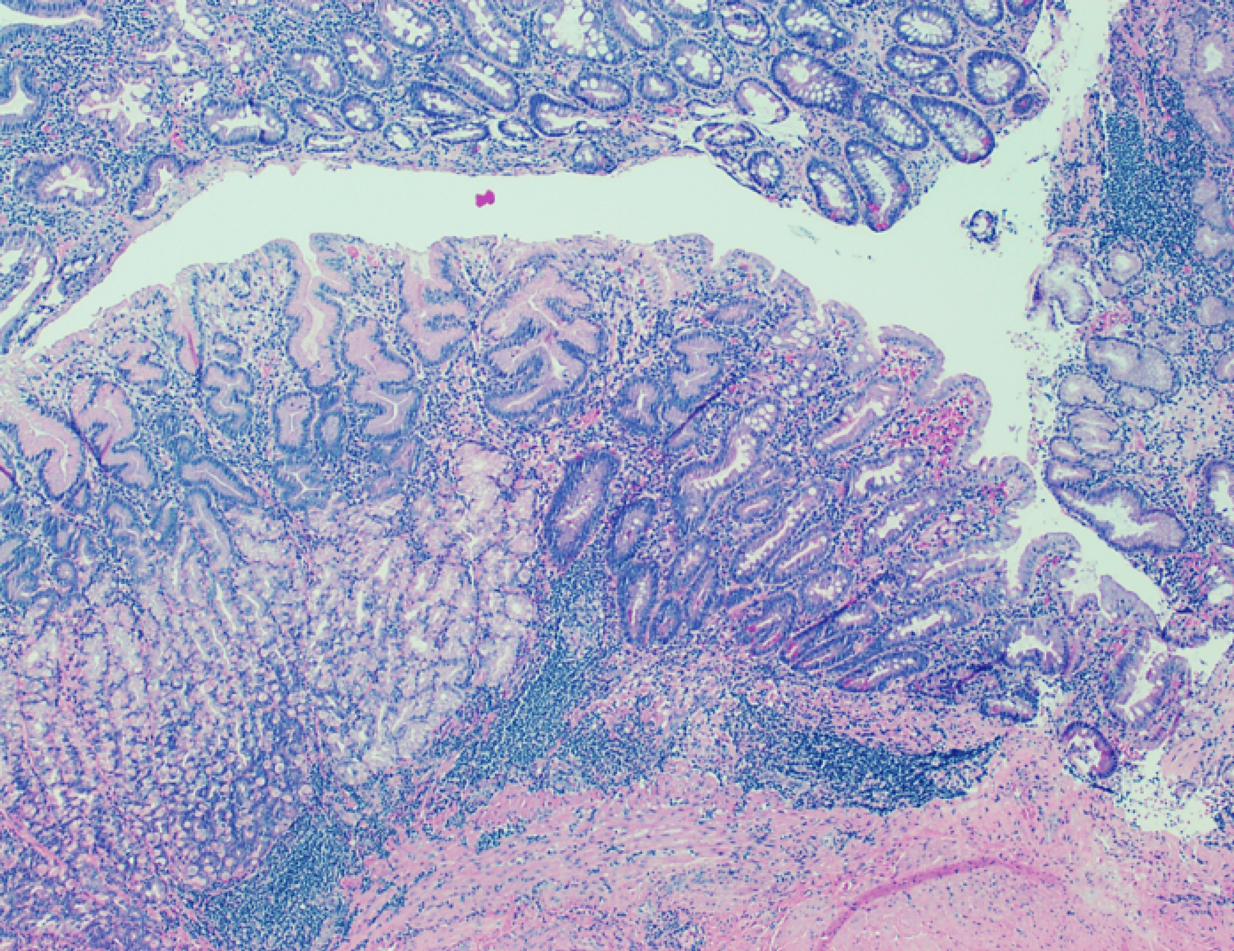
Gastric corpus: focal intestinal metaplasia (hematoxylin & eosin)

The histological result was benign in 19 patients (19/21); one patient was diagnosed with high-grade non-Hodgkin lymphoma, and only one patient had a diagnosis of adenocarcinoma (Figure 1). However, all the patients with benign histological results had some kind of alterations described in gastric tissue: five patients with necrosis, 13 patients with chronic gastritis (Figure 2), and two patients with erosive gastritis. Moreover, one patient also presented fungi colonization, and three patients presented with *H. pylori* bacillus (Figures 3-4). These patients were treated accordingly.

During follow-up (15 days to 15 months), 10 patients underwent UGE, and all had results consistent with the intraoperative biopsy. Two patients had malignancy detected previously, and eight patients with benign results underwent UGE.

In the group of patients with a benign histological result (n = 19), eight underwent UGE, with results excluding malignancy. Among the patients who did not undergo UGE (n = 11), three died postoperatively (from six hours to three days), one died on the 19th day postoperatively, one died from urosepsis during a second hospitalization after four months, one patient was lost to follow-up, in two patients UGE was not requested, and three patients are awaiting the procedure.

## Discussion

The treatment focus of PPU has always been the evasion of a hidden neoplasm, apart from damage control surgery. It is estimated that 2% to 5% of ulcers with a benign appearance are actually malignant. The diagnostic accuracy of the endoscopic technique and biopsy approaches is 99%, allowing the clinician to safely exclude the possibility of neoplasia [15]. Given this high diagnostic accuracy, should we still undergo IGUB in the endoscopic era?

In the case of PPU, biopsy of the ulcer margins is still routinely performed, despite recent guidelines from 2020 no longer recommending this approach. However, it is recommended if technically necessary for closure. It can be done if there is no healthy tissue available at the edge to suture or to approach the defect [1].

The literature describes several arguments in favor of IGUB and the methods for performing it, but studies regarding biopsy quality are scarce. Often, the patient's clinical course relies on the intraoperative biopsy, and it is not known whether this is reliable or if it provides a sufficient sample. Little is said about performing a biopsy on inflamed and damaged tissue, which could potentially be harmful. Moreover, it is believed that the quality of the sample obtained from UGE is superior to that taken intraoperatively [9].

Contrasting the high sensitivity of UGE for detecting malignancy with the low detection rate of gastric neoplasia using IGUB [14], this study should, at the very least, alert us and lead us to reflect on the true role of intraoperative biopsy. Despite this study detecting two cases of neoplasia early, both cases underwent repeated UGE, and the result was consistent with the intraoperative biopsy. This is because, despite the diagnosis, no clinician relies solely on that histological examination and needs to know what is happening with the rest of the gastric mucosa for treatment planning, in addition to performing further biopsies.

On the other hand, in cases where the intraoperative biopsy revealed benignity, the "modus operandi" dictates that UGE should be repeated to avoid missing any hidden neoplasm. This did not occur in all patients in this study, as only eight out of 19 patients underwent UGE, and all of them obtained the same result. However, six patients did not undergo it due to loss of follow-up or death, and three patients are still awaiting the exam. Thus, it can be concluded that only two out of 19 patients with benign histology did not undergo UGE during follow-up, by the attending physician's choice - a matter that is certainly debatable. The authors aim to raise awareness of possible therapeutic futility.

The limitations of the study include the small number of patients, which prevents us from recommending whether or not to perform IGUB, but rather only to question it. Additionally, during this seven-year period, we only included patients treated surgically via an open approach, so we did not study the results of laparoscopic or robotic biopsies. Furthermore, the rate of non-compliance during follow-up was considerable for the sample, which may compromise the results. Lastly, the retrospective nature of the study reduces its strength and could bias the results.

On the other hand, this study is strong enough to demonstrate that, at least, IGUB did not alter any clinical management in this sample. All the patients who underwent IGUB either repeated, should have repeated, or are going to repeat a histological biopsy of the gastric mucosa. The aim of our work was to raise awareness about possible futility in the treatment of these patients. Despite the limitations of the study, it is still possible to question the utility of IGUB in PPU.

## Conclusions

In the present study, performing IGUB did not alter the clinical course of any patient. In patients with malignant histology in the specimen, this was further confirmed endoscopically. In those with a benign specimen who underwent UGE, there were also no differences. In patients where IGUB was performed but no UGE was conducted during follow-up, clinical practice was incorrect, as there may be undetected hidden neoplasia. Thus, intraoperative ulcer biopsy proved to have no impact on the clinical course of the patients and should always be questioned.
